# Breast bruises and breast cancer

**DOI:** 10.1186/s13058-015-0631-y

**Published:** 2015-08-27

**Authors:** Nancy Krieger

**Affiliations:** Department of Social and Behavioral Sciences, Harvard T.H. Chan School of Public Health, Boston, MA 02115 USA

While recently examining one of the early classic works on breast cancer epidemiology—Janet E. Lane-Claypon’s 1926 report to the United Kingdom Ministry of Health [1]—and its 1931 US counterpart [2], I was struck by one item in the list of commonplace “antecedent conditions”: breast bruises, a not uncommon occurrence. Johannes Clemmensen’s classic review article in 1948 [3] also mention bruises to the breast as a possible etiologic factor.

In these articles, the discussion of bruises concerned how tissue trauma might increase risk of cancer [1–3]. As for the etiology of the bruises themselves? This was barely discussed, except with speculative reference to accidents and falls, whether at home or at work [1–3].

Any mention of breast bruises now, however, raises the question of whether intimate partner violence or other forms of physical abuse are at issue. A handful of new studies notably are investigating whether being physically or sexually abused acts as a psychosocial stressor that affects the risk of developing breast cancer (either directly or else indirectly, e.g., via increasing risk of obesity or alcohol consumption) [4] and also the likelihood of getting a mammogram [5]. As these studies attest, research on links between breast cancer and exposure to physical abuse and intimate partner violence remains scant [4, 5].

The historical juxtaposition is stark: (a) breast bruises having been routinely associated, in the past, with risk of breast cancer, albeit with no attention to violence as a possible cause [1–3] versus (b) intimate partner violence only recently becoming a focus of contemporary research on breast cancer [4, 5]. Although empirical data on long-term trends in violence involving breast bruises among women with breast cancer is likely unobtainable, nevertheless such violence is unlikely to be negligible [4, 5].

A lack of attention to breast bruises and violence in the current period is at best only partly explicable by changes in etiologic hypotheses regarding physical trauma as a cause of carcinogenesis.

The larger point is that a concern with breast cancer must be in the full context of the lives of those whom it affects, with due attention to the health problems to which breasts bear witness.
